# Simulation-Based Development, Stress Testing, and External Physiological Benchmarking of a DKA Early-Warning Framework Intended for Future Nurse-Facing Evaluation

**DOI:** 10.3390/healthcare14213790

**Published:** 2026-11-06

**Authors:** Muneera Alharbi

**Affiliations:** Department of Medical-Surgical Nursing, College of Nursing, Qassim University, Buraydah 52571, Saudi Arabia; mun.alharbi@qu.edu.sa

**Keywords:** diabetic ketoacidosis, nursing, machine learning, clinical decision support, early warning system, continuous ketone monitoring, beta-hydroxybutyrate, synthetic data, real-world data, alert fatigue

## Abstract

Background/Objectives: We evaluated a simulation-based diabetic ketoacidosis (DKA) early-warning framework and examined whether machine learning advantages reflected simulator assumptions. Methods: Twenty thousand synthetic 15 h episodes were generated at 5 min intervals. Validation-frozen beta-hydroxybutyrate (BHB) rules and XGBoost models were compared. Post hoc analyses assessed phenotype-specific glucose, counterfactual vital signs, comparator thresholds, and calibration. Public IOBP2 data were reconstructed for physiological benchmarking. Results: Among 449 reference-DKA test episodes, the original BHB+trend rule achieved 98.9% sensitivity and 1.060 false alerts/day; multimodal XGBoost achieved 89.5% and 0.456/day. At BHB ≥3.0 mmol/L, median glucose was 152.9 mg/dL in classical-DKA versus 304.5 mg/dL in non-DKA hyperglycemia, demonstrating a generator shortcut. With phenotype-identical vital signs, dual/multimodal AUPRC were 0.6044/0.6036; removing glucose and vital-sign phenotype cues yielded 0.5142/0.5164. An extended validation grid changed the BHB+trend cutoff and achieved 91.5% sensitivity at 0.761 false alerts/day. The adult IOBP2 benchmark contained 854 paired observations from 165 adults. Observed glucose medians exceeded synthetic medians by 108.7–243.4 mg/dL across five BHB strata. Conclusions: Conditional XGBoost discrimination depends on simulator construction and does not establish physiological superiority. IOBP2 exposes substantial glucose-distribution mismatch and does not validate DKA prediction or kinetics. Prospective clinical and nurse-facing assessment remains necessary.

## 1. Introduction

Diabetic ketoacidosis (DKA) remains one of the most serious acute metabolic complications of diabetes mellitus. DKA results from absolute or relative insulin deficiency accompanied by increased counter-regulatory hormone activity, leading to hyperglycemia, ketogenesis, dehydration, electrolyte abnormalities, and metabolic acidosis [1]. Although treatment outcomes have improved substantially, delayed identification can lead to severe metabolic disturbance, intensive-care requirements, and avoidable complications.

The 2024 international consensus report on hyperglycemic crises defines DKA through three components: diabetes or hyperglycemia, increased ketone concentration, and metabolic acidosis [1]. Blood beta-hydroxybutyrate (BHB) is particularly important because it is the predominant circulating ketoacid during DKA. A BHB concentration of ≥3.0 mmol/L correlates strongly with metabolic acidosis and is recommended for diagnosis and monitoring [1]. Marked hyperglycemia is not universally present; the consensus report notes that approximately 10% of patients with DKA may present with euglycemic DKA, an important phenotype in which glucose-centered surveillance may be misleading [1].

This limitation of glucose-centered surveillance becomes increasingly relevant as diabetes-monitoring technology evolves. Continuous glucose monitoring has transformed dynamic diabetes surveillance, while continuous ketone monitoring is moving from experimental development toward clinical implementation. Haas et al. demonstrated the feasibility of continuous interstitial BHB monitoring during DKA and reported strong agreement with venous measurements [2]. Continuous dual glucose–ketone monitoring has also been proposed as a strategy for recognizing emerging metabolic deterioration [3], and the Continuous Ketone Monitoring Consensus Panel outlined the potential role of continuous ketone sensing in DKA prevention and technology-enabled surveillance [4]. In August 2026, the U.S. Food and Drug Administration authorized the first wearable system capable of continuously measuring both glucose and ketones, further increasing the relevance of algorithms capable of interpreting combined trajectories [5].

Machine learning has been applied to DKA diagnosis and risk prediction using electronic health records [6,7,8]. More directly relevant, Cichosz and Bender analyzed 138 people with type 1 diabetes and more than 30,000 days of continuous-glucose-monitoring data to identify elevated ketone levels during hyperglycemia; their random-forest model achieved an AUROC of 0.836 [9]. In a later study of 259 participants with more than 49,000 monitoring days, integration of glucose and insulin-derived features yielded an AUROC of 0.82 and precision–recall AUC of 0.53 for elevated ketone detection [10]. These studies establish that short-horizon ketone-related prediction from real diabetes-monitoring data is feasible. Their endpoints, predictors, and sampling designs differ fundamentally from acid–base-defined DKA prediction, so their discrimination metrics should not be compared directly with the present synthetic framework.

Nursing surveillance represents a potential future use context because nurses integrate physiological measurements, trends, symptoms, behavioral changes, and clinical context to recognize deterioration and initiate escalation. Recent systematic reviews indicate that AI-supported clinical decision systems may strengthen nurses’ decision making, but robust nurse-centered evidence remains limited, particularly regarding workflow, alert burden, safety, and nurse-reported outcomes [11,12]. The CONCERN early-warning system demonstrated that ML models derived from nursing surveillance documentation can identify general patient deterioration and influence clinical outcomes [13]. The present study, however, did not include nurses, nursing documentation, usability testing, or clinical deployment. Accordingly, the revised framing is a DKA early-warning framework *intended for future nurse-facing evaluation*, rather than a validated nurse-centered system.

The intended future evaluation setting is adult inpatient acute-care surveillance (for example, ward or step-down care) in people with diabetes for whom ketone monitoring is clinically indicated. Continuous or high-frequency HR and RR streams make the current multimodal design more consistent with monitored inpatient care than with unsupervised home use. The present simulation does not estimate the size or prevalence of this target population.

Alert burden is a major consideration in clinical surveillance. Excessive or poorly targeted alerts can increase workload and reduce confidence in decision-support systems [14]. Recent work also emphasizes that alert fatigue is inconsistently operationalized and should not be inferred from alert counts alone [14]. Accordingly, the present study uses false alerts per simulated patient-day as an engineering burden metric, not as a validated measure of nurse alert fatigue.

Synthetic data provide a controlled environment for early algorithm development, but they cannot substitute for clinical validation. Synthetic diabetes data have been used for augmentation and for generation of continuous glucose-monitoring signals [15,16]. Such data permit deliberate construction of rare and ambiguous states, reproducible comparison of alert strategies, and stress testing before clinical deployment. To reduce the risk that the model simply learns a single simulator-defined trajectory shape, the present analysis uses multiple DKA and non-DKA BHB trajectory families with overlapping ketone levels and temporal patterns. Because physiological plausibility remains a central concern in simulation studies, a secondary analysis benchmarked selected glucose–ketone relationships and BHB temporal changes against independent public IOBP2 trial data previously used for real-world ketone modeling [10].

This study therefore developed and stress-tested a DKA early-warning framework using glucose, BHB, heart rate, respiratory rate, and recent temporal trends. pH and bicarbonate were simulated only to establish the hidden reference outcome and were not supplied to prediction models. The primary question was whether additional modeling complexity altered the trade-off between episode sensitivity, warning lead time, and alert burden relative to transparent continuous-ketone rules. The external-data analysis was prespecified as physiological benchmarking rather than external validation of DKA prediction because the public trial data contained intermittent, indication-driven ketone measurements and did not provide acid–base-confirmed pre-DKA endpoints.

The primary objective was to quantify the incremental value and limitations of ML relative to transparent BHB-based surveillance while jointly considering episode sensitivity, warning lead time, and false-alert burden. Secondary objectives were to compare glucose-only, glucose+BHB, and multimodal models; evaluate calibration sensitivity; use euglycemic DKA as a prespecified design stress test; assess transportability to a shifted synthetic cohort; and determine whether selected simulator assumptions were compatible with independently observed human glucose–ketone data. Additional post hoc analyses added during peer review were designed to quantify reference-definition circularity, assess conditional discrimination after BHB had already reached the diagnostic threshold, test dependence on HR/RR coupling and trajectory-family templates, and compare methods at matched operating points.

## 2. Materials and Methods

### 2.1. Study Design and Synthetic Cohort

This was a computational development, simulation-validation, and secondary public-data benchmarking study of a DKA early-warning framework intended for future nurse-facing evaluation. The primary model-development and stress-testing analyses used a physiologically constrained stochastic time-series simulator. No nurses or patients participated in system design, model development, or usability testing. A secondary analysis used de-identified, publicly available data from the Insulin-Only Bionic Pancreas Pivotal Trial (IOBP2; NCT04200313) solely to benchmark selected glucose–ketone and BHB-kinetic assumptions; no identifiable patient records were used.

The workflow consisted of synthetic episode generation, hidden reference labeling of DKA onset, temporal feature extraction, episode-level training/validation/test partitioning, Platt-calibrated model development, validation-only selection of model and rule operating points, independent testing, calibration sensitivity analysis, euglycemic-DKA stress testing, robustness testing, evaluation in a separately generated distribution-shift cohort, and an external real-world physiological benchmark performed after the primary simulation framework was frozen. Peer-review analyses were subsequently added as explicitly post hoc sensitivity analyses and did not alter the submitted primary cohort, labels, frozen models, or original operating points.

The study was designed with reference to TRIPOD+AI reporting principles for prediction-model studies [17]. DECIDE-AI informed the planned progression from in silico development to later human-factors and early clinical evaluation, although the present study does not constitute a clinical evaluation [18].

**Figure 1:**
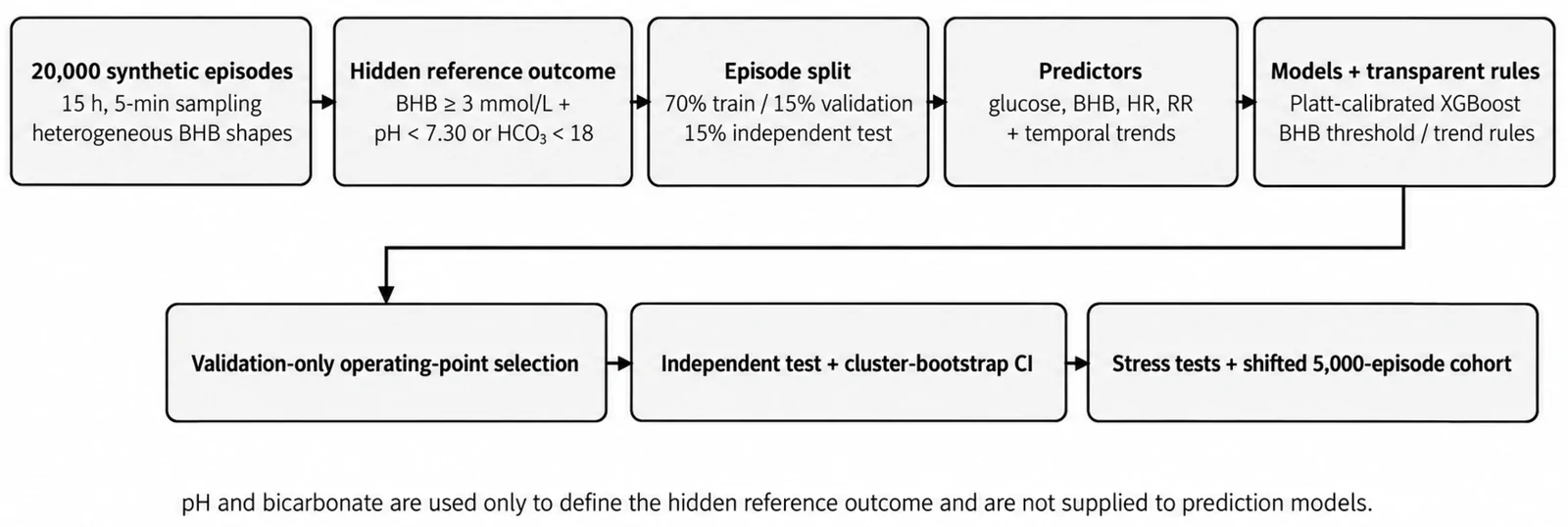
Study design and computational workflow. pH and bicarbonate are used only for hidden reference labeling and are not supplied to the prediction models.

The overall study workflow is summarized in Figure 1.

#### Synthetic Cohort

A total of 20,000 synthetic monitoring episodes were generated. Each episode represented a unique virtual adult with known diabetes and contained 15 h of observation sampled every 5 min. The assigned phenotype mix was 9000 stable diabetes episodes (45%), 5000 hyperglycemia episodes (25%), 3000 transient non-DKA ketosis episodes (15%), 2700 classical-DKA episodes (13.5%), and 300 euglycemic-DKA episodes (1.5%). Phenotype proportions were selected for algorithm development and do not represent clinical prevalence. Reference DKA status was determined after trajectory generation from the hidden BHB-acidosis criteria; no episode was forced across the reference threshold.

Table 1 summarizes the assigned synthetic phenotypes.

Euglycemic DKA represented 10% of the assigned DKA phenotype class. However, 682 of 2995 episodes fulfilling the hidden reference-DKA definition (22.8%) were euglycemic at onset; in the independent test partition, the proportion was 100/449 (22.3%). This exceeds the approximately 10% clinical figure cited above. Because the independently rising DKA BHB process often crossed the ketone threshold before the latent-severity-driven glucose trajectory had increased, some trajectories assigned to classical DKA met the euglycemic-at-onset criterion. The discrepancy is a generator artifact, not an estimate of clinical euglycemic-DKA prevalence.

### 2.2. Physiological Simulation and DKA Definition

#### 2.2.1. Latent Deterioration

For DKA-progressing episodes, metabolic deterioration was governed by a continuous latent severity variable S(t) ranging from 0 to 1. A logistic transition was used:

(1) $$S_{i}\left(t\right)=\frac{1}{1+\exp[-k_{i}\left(t-\tau_{i}\right)]}.$$

where *i* denotes the synthetic episode, $k_{i}$ is the patient-specific rate of deterioration, and $\tau_{i}$ is the midpoint of deterioration. Both parameters were sampled from prespecified distributions so that onset timing and rate of progression varied between episodes. For DKA-assigned episodes, glucose, heart rate (HR), respiratory rate (RR), bicarbonate, and pH were generated from this same latent severity process but with independently sampled amplitudes and variable-specific temporal offsets of up to 90 min. Thus, HR/RR and acid–base variables shared a latent deterioration driver in the primary simulator, although they were not forced to change synchronously. DKA-associated BHB was generated independently of S(t) with rise initiation sampled at 15–75 min, whereas the latent glucose and acid–base trajectory used a severity midpoint of approximately 300–720 min and variable-specific offsets up to 90 min. Consequently, BHB frequently crossed 3.0 mmol/L before a glucose rise. This misalignment can invert the clinically anticipated glucose–ketone relationship; the present study does not claim that the resulting high-BHB discrimination generalizes to patients.

#### 2.2.2. Simulated Physiological Variables

Six physiological variables were generated: glucose, BHB, heart rate, respiratory rate, bicarbonate, and pH. Only glucose, BHB, heart rate, and respiratory rate were available to the prediction models. Bicarbonate and pH were hidden reference variables used exclusively to determine whether and when simulated DKA criteria were met.

Baseline values were sampled from glucose 90–180 mg/dL, BHB 0.10–0.55 mmol/L, heart rate 62–92 beats/min, respiratory rate 12–18 breaths/min, bicarbonate 23–28 mmol/L, and pH 7.36–7.44. For DKA-assigned episodes, BHB typically began at 0.25–0.75 mmol/L, and post-delay rise rates were sampled predominantly from 0.18–0.35 mmol/L/h. Klonoff et al. reported an extracted mean BHB increase of 0.0038 mmol/L/min after pump interruption (approximately 0.20 mmol/L/h as rounded in the published abstract), and an estimated mean time of 14.2 h to BHB 3.0 mmol/L, with severe ketonemia across clinical studies estimated at 13–21 h [19]. The simulation interval was a modeling choice rather than an independently validated human DKA-rate distribution. Its reference-onset median of 645 min (10.75 h) is faster than the pump-interruption time anchor, even though biochemical DKA onset and ketone-only thresholds are not identical endpoints. The HR and RR increment ranges in Table 2 were likewise modeling ranges rather than fitted clinical effect sizes. Table 2 lists the modeled physiological variables and assumptions.

#### 2.2.3. Trajectory Generation and Stochastic Variation

For glucose, heart rate, respiratory rate, bicarbonate, and pH, episode-specific trajectories combined a latent deterioration process with independently sampled amplitudes, temporal offsets, and correlated stochastic variation. DKA-associated BHB was generated independently of the latent logistic curve to avoid a single deterministic coupling between all physiological channels.

DKA-assigned BHB trajectories were sampled from five families: linear rise, two-stage rise, pause-and-resume, accelerating rise, and variable ramp. Rates varied within and, for the variable-ramp family, locally around the empirically anchored range. Non-DKA transient ketosis was sampled from six families: Gaussian bump, ramp-to-plateau, ramp followed by reversal, two-stage plateau, pause-then-recovery, and multi-bump. Hyperglycemia episodes could also show slow BHB ramps or sigmoid plateaus. These choices intentionally created early rising patterns in non-DKA episodes that could resemble DKA before later plateau or reversal.

Non-DKA acid–base trajectories were constrained to remain outside the reference DKA definition, while BHB itself was allowed to exceed 3 mmol/L. The generator uses code-level non-DKA floors of bicarbonate 18.2 mmol/L and pH 7.305 unless explicitly overridden through the corresponding non-DKA floor keys; these values are immediately outside the DKA reference thresholds and create hard negatives by construction. The frozen configuration also contains unused legacy transient-ketosis floor settings (18.5 mmol/L and pH 7.31) and a legacy bhb_rise_mmol_l field; these are not active control variables in the implemented generator and are documented as such in the updated code archive. The resulting cohort therefore contained substantial overlap in current BHB concentration and recent trajectory shape between reference-DKA and non-DKA states.

(2) $$\epsilon_{t}=\rho \epsilon_{t-1}+\eta_{t}.$$

Temporal noise followed a first-order autoregressive process with coefficient ρ=0.72. In Equation (2), $\eta_{t}$ denotes a zero-mean Gaussian innovation, $\eta_{t}∼N\left(0,\sigma^{2}\right)$, with variable-specific standard deviation. Innovation sequences were sampled independently across physiological channels and episodes. Innovation standard deviations were 8.0 mg/dL for glucose, 0.12 mmol/L for BHB, 2.8 beats/min for heart rate, 1.1 breaths/min for respiratory rate, 0.45 mmol/L for bicarbonate, and 0.009 for pH. These values were simulation assumptions rather than estimates of a specific sensor or patient population.

#### 2.2.4. Non-DKA Trajectories

Stable diabetes: glucose and BHB fluctuated around episode-specific baselines with correlated stochastic variation, while vital signs and acid–base variables remained stable.

Hyperglycemia without DKA: glucose could rise substantially; BHB could increase as a slow ramp or sigmoid plateau to as high as 3.4 mmol/L, accompanied by variable heart-rate and respiratory-rate changes, while pH and bicarbonate remained outside the reference DKA criteria.

Transient ketosis without DKA: BHB peaks were sampled from 0.8–4.8 mmol/L and could follow DKA-like rising trajectories that plateaued, paused, or reversed. Glucose and vital signs were only loosely coupled to ketone burden, and mild acid–base changes were permitted while remaining outside the DKA reference definition.

These overlapping hard-negative states were included specifically to prevent either a BHB concentration threshold or a single ramp-versus-bump trajectory shape from deterministically defining the target.

#### 2.2.5. Reference DKA Definition

Reference DKA onset was derived from the 2024 international consensus criteria [1]. Because every virtual patient had pre-existing diabetes, the diabetes/hyperglycemia component was considered fulfilled by history. The first time point satisfying both BHB ≥ 3.0 mmol/L and pH <7.30 and/or bicarbonate <18 mmol/L was defined as DKA onset. Glucose was therefore not required to exceed 200 mg/dL, allowing euglycemic DKA to be represented. pH and bicarbonate were not used as predictors. Prediction ceased at the reference onset; observations at or after established DKA were excluded from the early-warning target.

### 2.3. Prediction Target, Predictor Variables, and Feature Engineering

At each eligible 5 min time point, the model estimated the probability that the episode would meet reference DKA criteria during the following 120 min. The primary prediction horizon was 2 h. Secondary analyses used 30, 60, and 120 min horizons. An observation was labeled positive if DKA onset occurred within the corresponding future horizon and negative otherwise.

#### 2.3.1. Predictor Sets

Four prespecified predictor configurations were examined: glucose-only temporal features; glucose+BHB temporal features; a multimodal set adding heart rate and respiratory rate; and a simple ketone model containing only current BHB and 30 min BHB slope. pH, bicarbonate, anion gap, diagnostic labels, and treatment variables were excluded from all predictors.

The predictor configurations are summarized in Table 3.

#### 2.3.2. Feature Engineering

Temporal slopes were estimated by ordinary least-squares regression of each variable against time over the preceding 30 min. Rolling means were calculated over the preceding 60 min. Features were calculated using only information available at or before the index time to prevent future-data leakage. Continuous features were standardized using parameters derived exclusively from the training partition when required by the algorithm.

### 2.4. Models, Calibration, and Data Partitioning

XGBoost was used for glucose-only, glucose+BHB, and multimodal predictor sets. A two-feature logistic-regression model using current BHB and the 30 min BHB slope provided an interpretable parametric comparator. The final hyperparameters were fixed in the frozen config_final.yaml before the final v4 analysis; no automated hyperparameter search was performed on the independent test set. These settings are therefore treated as development choices rather than optimized clinical estimates. Model probabilities were calibrated using Platt/sigmoid calibration fitted only on the validation partition; isotonic calibration was retained as a sensitivity analysis.

The frozen modeling settings are reported in Table 4.

The final analysis and peer-review checks were reproduced under Python 3.13.5 with NumPy 2.3.5, pandas 2.2.3, SciPy 1.17.0, scikit-learn 1.8.0, XGBoost 3.1.3, SHAP 0.50.0, Matplotlib 3.10.8, and joblib 1.5.3. The global simulation/model seed was 20260911. The complete configuration and scripts are supplied with the reproducibility package.

#### 2.4.1. Dataset Partitioning and Prevention of Leakage

Episodes, rather than time points, were stratified by assigned phenotype and allocated to training (70%), validation (15%), and independent test (15%) partitions. All time points from one episode remained in a single partition. Because reference DKA status was determined after trajectory generation rather than forced by assigned phenotype, the realized number of reference-DKA episodes could differ slightly from the assigned number. No model fitting, calibration, operating-point selection, or hyperparameter selection used the independent test set.

#### 2.4.2. Class Imbalance, Probability Calibration, and Missing Data

Impending-DKA time points represented a minority of observations. Class imbalance was addressed using class weighting within model training; time-point-level synthetic oversampling was avoided because it could distort temporal relationships. AUPRC was selected as a primary performance metric because it is more informative than accuracy when positive events are uncommon.

Platt/sigmoid calibration was fitted exclusively on the validation partition and used for the primary analysis; isotonic calibration was evaluated as a sensitivity analysis. Calibration was assessed using the Brier score, calibration intercept, calibration slope, and calibration plots. For the complete-data primary analysis, feature rows were eligible only after a complete 60 min history was available, so rolling means, slopes, and deltas were not calculated across missing raw values. In missingness stress tests, corrupted raw predictor streams were forward-filled within episode for at most 15 min (three 5 min samples); remaining missing raw values were replaced by medians from the corrupted test copy before temporal features were recalculated. Model pipelines also contained median imputation with missingness indicators as a final safeguard.

### 2.5. Comparator Strategies and Alert Definition

Three transparent ketone comparators were evaluated. A current-BHB threshold was selected from a prespecified validation grid. A BHB+positive-trend AND rule required both current BHB to exceed a floor and the 30 min BHB slope to meet a minimum positive cutoff; because a zero slope cutoff was included in the grid, this rule could reduce to a simple threshold if the slope term provided no benefit. Finally, a logistic model used only current BHB and 30 min BHB slope. For every comparator and probability model, the operating point was chosen exclusively on validation data as the option maintaining at least 90% episode-level sensitivity while minimizing false alerts per simulated patient-day, with warning lead time used only as a secondary tie-breaker. The selected rule parameters and probability thresholds were then frozen before independent testing; test sensitivity was therefore permitted to fall below 90%.

The primary alert analysis used one binary alert threshold per strategy. An alert was issued when the score was at or above threshold and either crossed from below threshold or at least 60 min had elapsed since the preceding alert from that strategy. Thus, the earlier wording “same or higher level” did not apply to the primary binary analysis and has been removed. An alert was classified as a true early warning when reference DKA developed within the subsequent 120 min. Under the prespecified primary definition, alerts more than 120 min before eventual DKA onset were counted as false alerts because they fell outside the prediction horizon. Total monitored patient-days in the denominator included non-DKA observation time and pre-onset observation time from DKA episodes; post-onset time was excluded.

*False alerts per patient-day = number of false alerts/total monitored pre-onset or non-DKA patient-days.*

A post hoc sensitivity analysis added during peer review separately classified alerts occurring more than 120 min before eventual DKA as “early precursor alerts” rather than false alerts, while leaving 120 min episode sensitivity unchanged.

### 2.6. Intended Future Nurse-Facing Evaluation Context

The original submission included a conceptual Green/Yellow/Orange/Red display. Because no nurses participated in design or evaluation and the display had not undergone usability testing, the categorical interface table has been removed from the revised manuscript. The present study evaluates risk scores and a single binary operating threshold only; it does not propose a validated clinical protocol, treatment recommendation, or universal display cutoff. Future interface development would require co-design and human-factors evaluation with nurses and other clinical stakeholders in the intended inpatient setting.

### 2.7. Stress Tests and Explainability

Euglycemic DKA was prespecified as a design stress test rather than a discovery analysis. The subgroup comprised reference-DKA episodes with glucose <200 mg/dL at onset, regardless of assigned DKA phenotype. The purpose was to test the expected vulnerability of glucose-centered alerting when marked hyperglycemia was absent.

Robustness analyses introduced 20% random missingness, a 30 min contiguous data gap, and additional measurement noise in the independent test partition. A separately generated distribution-shift cohort of 5000 episodes was evaluated without retraining or reselecting thresholds. This cohort used an 18 h monitoring window, slower DKA BHB rise rates (0.15–0.30 mmol/L/h), longer BHB delays (30–120 min), temporal offsets up to 120 min, 35% greater stochastic noise, hyperglycemic BHB values up to 3.6 mmol/L, and transient non-DKA peaks up to 5.0 mmol/L.

#### Explainability Analysis

Shapley additive explanations (SHAP) were calculated for the final multimodal XGBoost model. Global importance was summarized using mean absolute SHAP values. Because the data are synthetic, SHAP values were interpreted only as descriptions of model behavior within the imposed data-generating process and not as causal clinical importance.

### 2.8. Post Hoc Sensitivity Analyses Added During Peer Review

The following analyses were added after peer review and did not alter the frozen primary analysis. First, reference-definition circularity was quantified by identifying, for each reference-DKA episode, whether BHB ≥3.0 mmol/L or the acid–base criterion was the later component and therefore set the reference onset time. Discrimination was also recalculated after restricting eligible test observations to current BHB ≥3.0 mmol/L; a separate hard-negative analysis examined non-DKA observations already above this BHB threshold.

Second, we retained the initial HR/RR temporal-decoupling analysis for historical reproducibility, but recognized that it preserved phenotype-specific vital-sign signatures. We therefore added counterfactual retraining with HR/RR drawn identically across all phenotypes, with glucose driven by an independent temporal process, and with both changes combined. These explicitly exploratory model refits do not replace the original frozen test evaluation; their purpose is to identify model dependence on generator-specific predictors.

Third, test-set episode sensitivity versus false-alerts/day curves were calculated across operating thresholds for all five BHB-based strategies. Methods were compared at matched operating conditions: ML burden at the sensitivity achieved by the transparent rules and rule sensitivity at the submitted multimodal false-alert burden. Differences between submitted operating points were additionally estimated with 2000 paired episode-cluster bootstrap replicates so that both methods in each comparison used the same resampled episodes.

Fourth, performance was stratified by BHB trajectory family, and a leave-one-family-out analysis excluded each DKA or non-DKA family from both training and validation before retraining and testing on the unseen family. Fifth, false-alert rates were decomposed by assigned phenotype, the DKA-onset-time distribution was summarized, and the primary alert definition was compared with the early-precursor sensitivity definition described above. Additional second-round analyses tabulated the full glucose distributions in BHB ≥3 observations by assigned phenotype, reported hard-negative episode-level threshold exposure, extended the validation search grid for the BHB+trend comparator, and assessed risk-spaced calibration in the full test sample and the BHB ≥3 conditional subset. Extended-grid settings were selected on validation and evaluated on test only as sensitivity analyses. Matched operating points were located on the test set and are descriptive, post hoc comparisons, not independent validation of reselected thresholds.

### 2.9. IOBP2 Data Reconstruction and Benchmarking Methods

A secondary physiological benchmarking analysis used the public IOBP2 randomized-trial dataset [20,21], the same source subsequently reanalyzed for ketone-prediction research [10]. The trial enrolled adults and children with type 1 diabetes and collected Dexcom G6 continuous glucose monitoring (CGM) together with capillary blood ketone measurements in the bionic-pancreas treatment arms. Because ketone testing was clinically prompted, particularly during hyperglycemia or suspected infusion-set failure, these observations were not treated as a prevalence sample and were not used to estimate DKA prediction performance.

The publicly available SAS7BDAT-format ketone, CGM, and participant-roster tables were read using Python (pyreadstat), without using SAS statistical software, and linked by participant ID and study-recorded device timestamps. Adults aged ≥18 years with a roster treatment code BP or BPFiasp were included. For each ketone observation, the *latest preceding* true CGM reading (excluding calibration records) was required to fall within ten minutes, and at least 72 CGM readings were required during the preceding 12 h. No timezone adjustment was imposed. The reconstructed cohort reproduced 854 paired observations from 165 adults (452 observations from 84 BP participants and 402 from 81 BPFiasp participants); 157 pairs had BHB ≥0.6 mmol/L, and 18 had BHB ≥3.0 mmol/L. Across all ages in BP/BPFiasp, identical criteria yielded 1799 pairs from 258 participants. These eligibility rules were audited before examining synthetic–real differences.

Five BHB strata (<0.6, 0.6–<1.0, 1.0–<1.5, 1.5–<3.0, and ≥3.0 mmol/L) were compared. The primary frozen synthetic comparator regenerated all 20,000 episodes (train, validation, and test partitions) and included non-DKA observations and only *pre-onset* rows from episodes that met the reference-DKA criteria: 3,466,827 synthetic rows. The restricted comparator included 389,827 such pre-onset rows from all 3000 episodes assigned to classical/euglycemic DKA, regardless of eventual reference status. Because the data-generating populations and sampling rules differ, this is a descriptive model-design audit, not an independent external validation.

The comparisons report observed and synthetic medians, their differences, and a Wasserstein-type distance computed using the recorded capped CGM values as a *descriptive proxy*, without claiming an uncensored estimate. Readings of ≥401 mg/dL were treated as right-censored at the CGM reporting ceiling. Participant-clustered bootstrap intervals (2000 resamples, resampling adult IDs with replacement) quantified uncertainty in observed-minus-fixed-synthetic median differences. Participants per stratum and counts at the CGM ceiling were reported. A sensitivity comparison repeated the strata restricted to DKA-assigned synthetic episodes.

All adult serial BHB differences from the reconstructed cohort separated by 0.5–6 h were retained (positive, negative, and zero slopes), grouped by elapsed-time interval, and summarized with participant counts and median signed slopes. The dataset did not identify acid–base-confirmed DKA trajectories, so these slopes describe *non-DKA ketone fluctuation*, not pre-DKA progression. The assay’s measurement-specific error specification was unavailable; therefore, a numerical uncertainty floor was not estimated from this cohort. Instead, an explicit assumed absolute single-reading uncertainty δ was used for illustrative sensitivity bounds |ΔBHB|/Δt≤2δ/Δt (*not* an empirical assay performance claim). The trial’s principal randomized report found no DKA events [20]; no prediction performance was estimated using IOBP2.

### 2.10. Outcomes, Statistical Analysis, and Ethics

The primary outcomes were AUPRC, episode sensitivity at the selected operating threshold, median warning lead time, and false alerts per patient-day. Warning lead time was defined as the interval between the first qualifying alert within the 120 min horizon and reference DKA onset among correctly identified DKA episodes. Secondary outcomes were AUROC; specificity; positive predictive value; negative predictive value; F1 score; Brier score; calibration slope and intercept; sensitivity for euglycemic DKA; performance at 30, 60, and 120 min horizons; and performance under missing measurements and simulated sensor noise.

Uncertainty in the frozen primary analysis was estimated by episode-cluster resampling: 200 replicates (seed 20260912) for time-point discrimination and Brier-score intervals and exactly 500 replicates for episode-level alert outcomes. In the revised calibration table, calibration intercept and slope receive 95% episode-cluster-robust confidence intervals. The peer-review matched-operating-point comparisons used 2000 paired episode-cluster bootstrap replicates. Comparisons were interpreted as design-performance trade-offs within the synthetic environment rather than evidence of clinical superiority.

#### Ethical Considerations

The primary development analysis used computationally generated synthetic data. The secondary benchmark used de-identified public IOBP2 data from a previously approved clinical trial [20,21]. No new participants were recruited, and no identifiable information was accessed. The original IOBP2 trial obtained institutional review board approval and informed consent/assent as applicable [20].

## 3. Results

### 3.1. Synthetic Cohort Characteristics and Reference-Onset Decomposition

All 20,000 assigned episodes were generated as prespecified. Of 3000 episodes assigned to classical or euglycemic DKA phenotypes, 2995 met the hidden reference-DKA definition within the 15 h observation window; no reference label was forced. The hard-negative design produced 49,874 non-DKA time points with BHB ≥3.0 mmol/L. The independent test partition contained 3000 episodes and 449 reference-DKA episodes. In the separately generated 5000-episode shifted cohort, 748 episodes met the reference-DKA definition; no episode was forced across the criteria.

The post hoc onset-component analysis quantified the reference-definition circularity. Among the 2995 main-cohort reference-DKA episodes, BHB was the later criterion and therefore set reference onset in 1854 episodes (61.9%), the acid–base criterion was later in 1107 (37.0%), and both were first satisfied at the same 5 min sample in 34 (1.1%). In the independent test set, reference DKA onset ranged from 420 to 895 min, with a median of 645 min (IQR 585–710 min), indicating that the 120 min warning horizon was not generally truncated by very early onset.

The main cohort included multiple BHB trajectory families in both DKA and non-DKA states, including linear, accelerating, two-stage, pause/resume, plateau, reversal, and multi-bump patterns.

**Figure 2:**
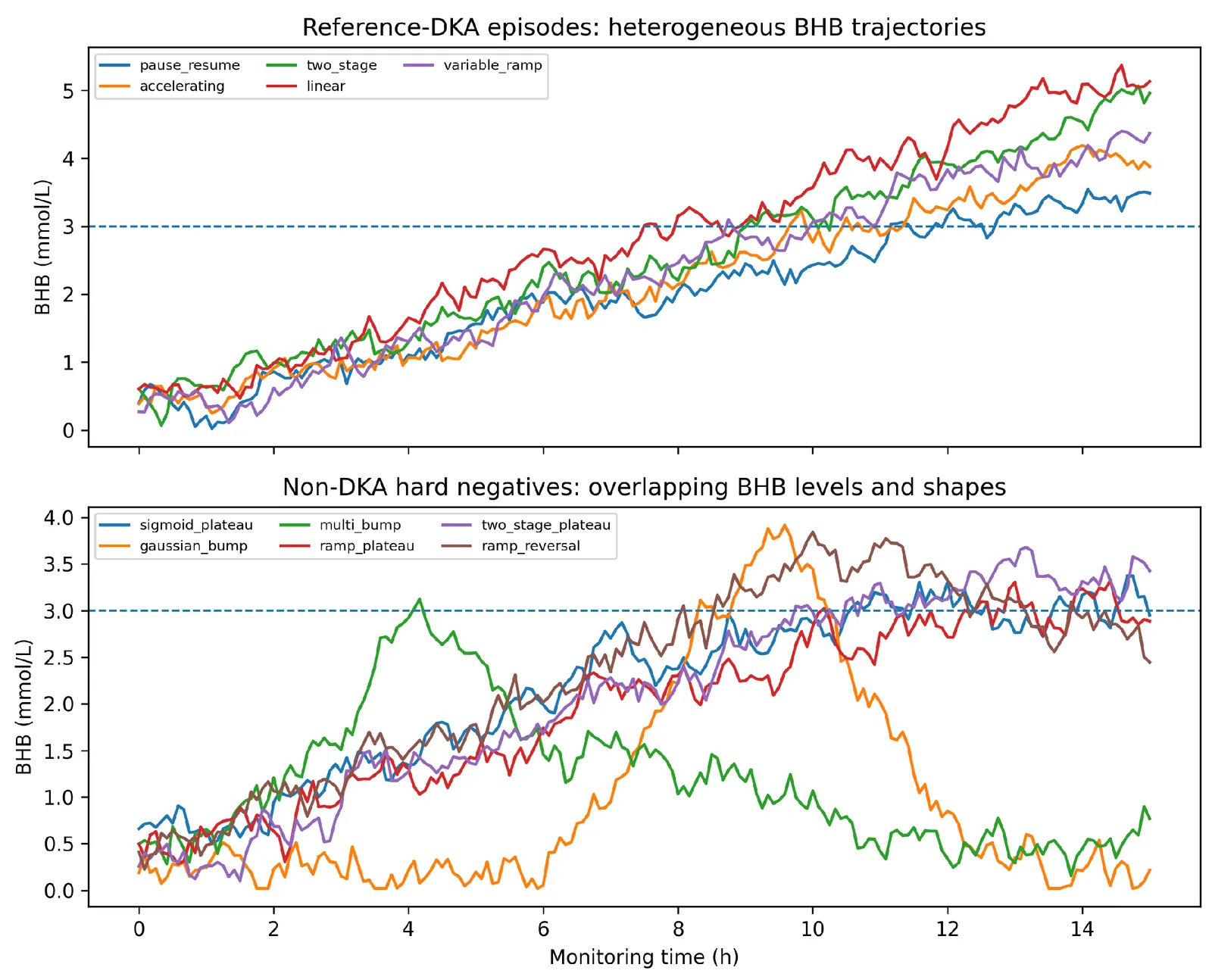
Examples of heterogeneous BHB trajectories in reference-DKA episodes and non-DKA hard negatives. The blue dashed horizontal lines in both panels mark the BHB reference threshold of 3.0 mmol/L. Non-DKA trajectories can exceed 3 mmol/L and can display sustained early rises before plateau or reversal.

Representative DKA and non-DKA BHB trajectories are illustrated in Figure 2.

### 3.2. Overall Model Performance and Conditional High-BHB Analysis

On the independent test set, multimodal XGBoost achieved AUROC 0.989 (95% CI 0.988–0.990), AUPRC 0.657 (95% CI 0.630–0.695), and Brier score 0.0117 (95% CI 0.0107–0.0127). Dual glucose+BHB XGBoost achieved AUROC 0.987 (95% CI 0.985–0.988) and AUPRC 0.604 (95% CI 0.569–0.640). Glucose-only XGBoost achieved AUROC 0.758 and AUPRC 0.105, while the two-feature BHB-trend logistic model achieved AUROC 0.969 and AUPRC 0.262.

**Figure 3:**
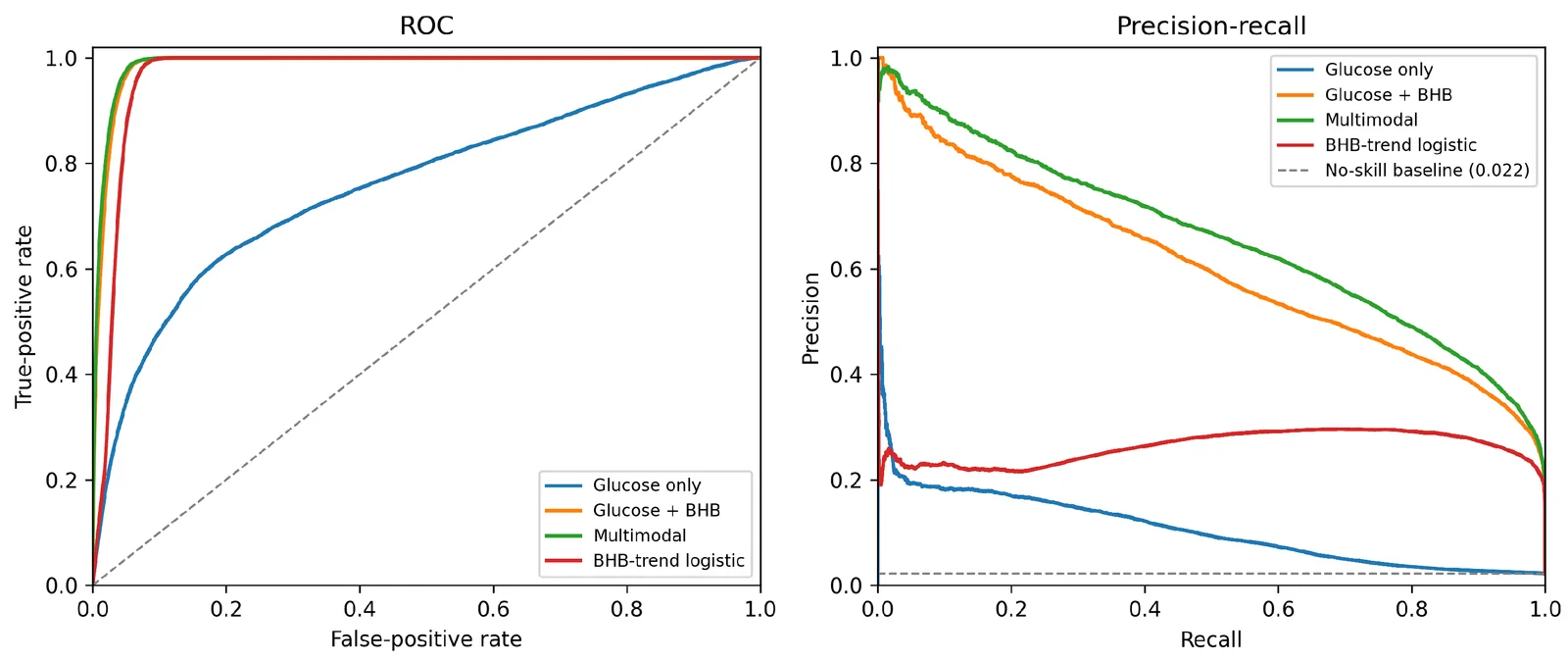
Time-point-level receiver-operating-characteristic (ROC; left) and precision-recall (right) curves in the independent test set. The gray dotted diagonal line in the ROC panel represents chance-level discrimination (true-positive rate = false-positive rate; AUROC = 0.5). The gray dashed horizontal line in the precision-recall panel indicates the no-skill baseline at the observed time-point positive-event prevalence (0.0223).

Independent-test discrimination curves are presented in Figure 3.

**Figure 4:**
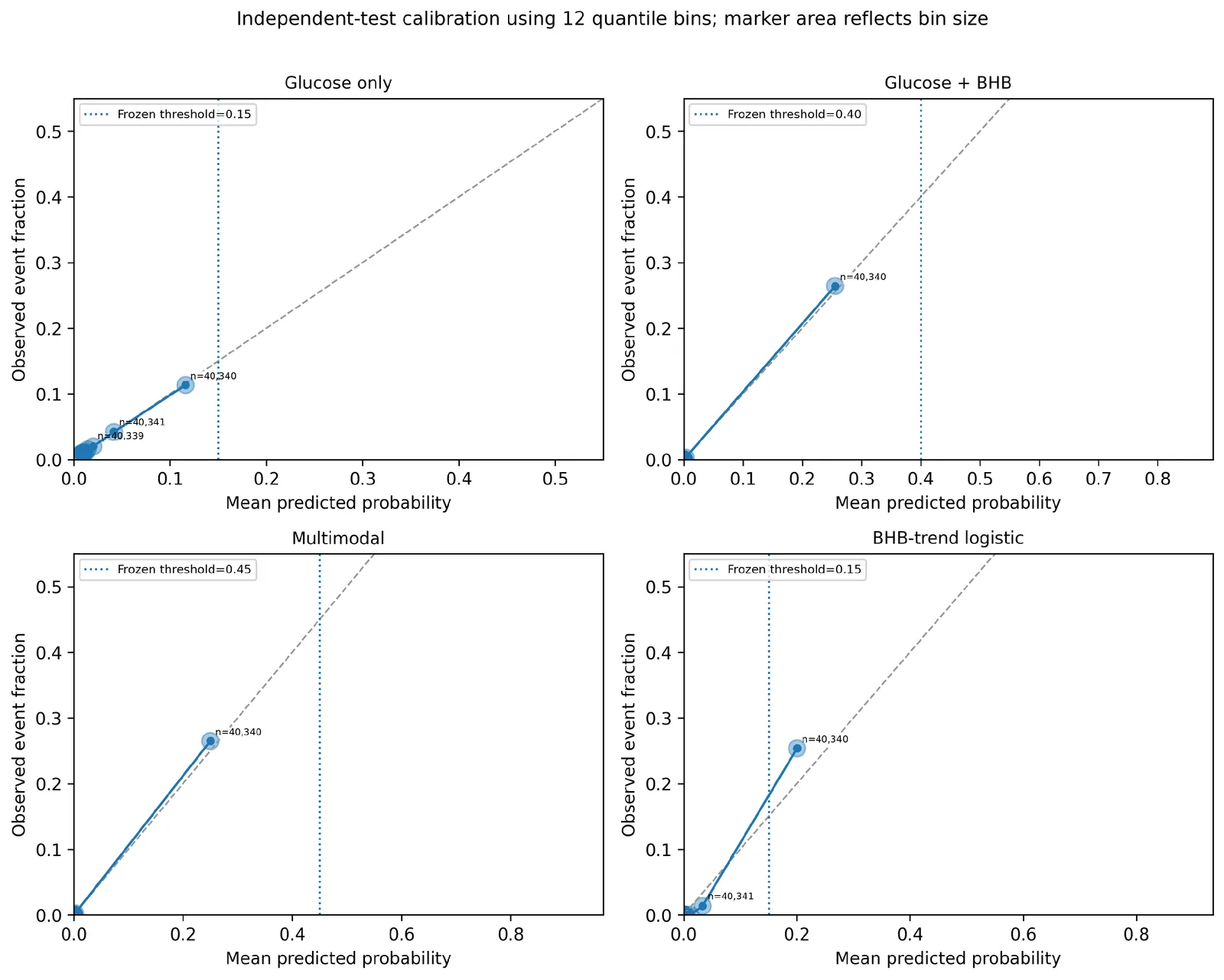
Independent-test calibration plots using 12 quantile bins for each model. The gray dotted diagonal line represents perfect calibration (observed event fraction = mean predicted probability). The blue solid line connects the observed event fractions across the predicted-probability bins. Marker areas and adjacent labels indicate the number of observations in each bin. Vertical dotted lines indicate the model-specific operating thresholds frozen on the validation set.

The original quantile-binned calibration displays are shown in Figure 4.

Model discrimination and calibration metrics are reported in Table 5.

#### 3.2.1. Conditional Performance and the Glucose-Generation Inversion Above BHB 3.0 mmol/L

Eligible test observations with current BHB ≥3.0 mmol/L included 10,265 rows (2259 impending-DKA positives and 8006 negatives). The BHB-trend logistic model had AUROC 0.509 and AUPRC 0.228, versus dual XGBoost AUROC/AUPRC 0.882/0.669 and multimodal 0.921/0.725. These results must *not* be interpreted as evidence of clinically meaningful information beyond a BHB threshold. In the frozen generator, median glucose at BHB ≥3 was much lower in classical- and euglycemic-DKA branches (152.9 and 149.8 mg/dL) than in non-DKA hyperglycemia (304.5 mg/dL) and transient ketosis (196.0 mg/dL); a tree model can exploit this branch distinction. Table 6 documents distributions stratified by phenotype, including the non-DKA hard negatives.

The phenotype-specific high-BHB glucose distributions are detailed in Table 6.

Among 7296 BHB-high non-DKA hard-negative rows (241 episodes), the multimodal model crossed its threshold in 5.10% of rows but in 43/241 episodes (17.8%); dual XGBoost crossed in 8.13% of rows and 81/241 episodes (33.6%); the BHB+trend rule crossed in 50.04% of rows and 239/241 episodes (99.2%). These are episode-level exposures to *at least one score crossing*, not refractory-adjusted alert counts.

#### 3.2.2. Vital-Sign and Glucose Counterfactuals

The prior HR/RR temporal-decoupling sensitivity analysis lowered multimodal AUPRC from 0.657 to 0.647, but preserved phenotype-specific vital-sign signatures and could not test whether the residual increment was a simulator shortcut. With identically generated HR/RR across all phenotypes, dual/multimodal AUPRC were 0.6044/0.6036. With glucose independently generated but original vital-sign families retained, corresponding AUPRC were 0.5221/0.6056. When both glucose and vital-sign phenotype cues were removed, dual/multimodal AUPRC were 0.5142/0.5164. These results directly demonstrate that the original incremental discrimination depends on the simulator’s phenotype-dependent construction; they do not provide biological evidence for added predictor value.

### 3.3. Early-Warning Performance, Matched Operating Points, and Alert Definition Sensitivity

At the validation-frozen operating points, the BHB threshold detected 444/449 reference-DKA episodes (98.9%; 95% CI 97.8–99.8%) with a median 75 min warning lead time and 1.193 false alerts/day. The BHB+positive-trend AND rule used a BHB floor of 2.75 mmol/L and slope cutoff of 0.004 mmol/L/min (0.24 mmol/L/h); it also detected 444/449 episodes with 1.060 false alerts/day. The BHB-trend logistic model detected 443/449 (98.7%) with 1.192 false alerts/day. Dual glucose+BHB XGBoost detected 412/449 (91.8%) with 0.624 false alerts/day and an 80 min median lead time. Multimodal XGBoost detected 402/449 (89.5%) with 0.456 false alerts/day and an 85 min median lead time. Glucose-only XGBoost reached 354/449 episodes (78.8%) at the lowest available probability threshold, 0.05, with 4.890 false alerts/day, and did not meet the intended 90% validation sensitivity target. These frozen results represent different operating points on different sensitivity–burden curves and therefore do not establish that one strategy uniformly dominates another.

The primary frozen alert metrics are summarized in Table 7.

#### 3.3.1. Matched Operating-Point Analysis

**Figure 5:**
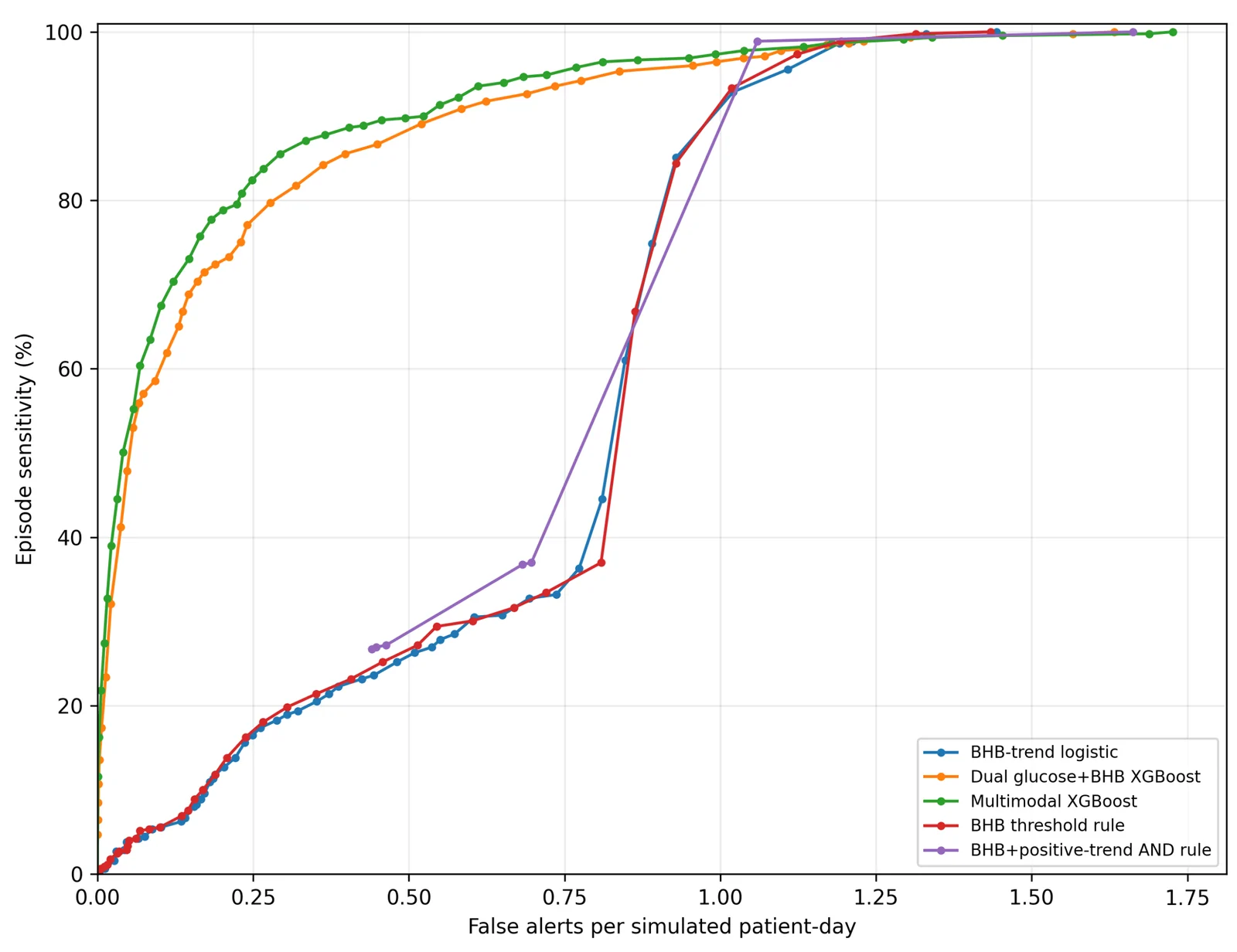
Independent-test episode sensitivity versus false alerts per simulated patient-day across thresholds for the BHB-trend logistic model, dual glucose+BHB XGBoost, multimodal XGBoost, BHB threshold rule, and BHB+positive-trend AND rule. Curves demonstrate that the submitted operating points represent different locations on a common sensitivity–alert-burden frontier.

Figure 5 places all five strategies on common test-set episode-sensitivity versus false-alert curves. At the transparent-rule sensitivity of 98.89%, the closest dual-XGBoost operating point produced 1.230 false alerts/day, and the closest multimodal point produced 99.11% sensitivity with 1.295 false alerts/day. Conversely, when transparent rules were constrained to no more than the submitted multimodal burden of 0.456 false alerts/day, the BHB threshold achieved 23.2% sensitivity (0.407/day), and the BHB+trend rule achieved 26.9% (0.447/day). At matched high sensitivity, the dual and multimodal models both had median lead times of 95 min, compared with 70 min for the BHB-trend logistic comparator. These operating points were selected retrospectively on the test set and are *descriptive only*; they are not unbiased estimates of validation-frozen operating points. Thus, the low submitted ML burden was not retained when ML was moved to the rules’ very-high-sensitivity region, and the rules could not retain high sensitivity when moved to the multimodal burden region.

Paired episode-cluster bootstrap comparisons at the submitted points quantified the same trade-off. Relative to multimodal XGBoost, the BHB+trend rule had 9.35 percentage points higher sensitivity (95% CI 6.57–12.53) and 0.603 more false alerts/day (95% CI 0.513–0.703). Dual XGBoost had 2.23 percentage points higher sensitivity than multimodal (95% CI −0.49 to 5.11) and 0.168 more false alerts/day (95% CI 0.113–0.227).

#### 3.3.2. Extended-Grid Transparent Comparator Sensitivity

The original BHB+trend slope selection of 0.004 mmol/L/min was at the top of its candidate grid. Extending that grid selected a BHB floor of 2.75 mmol/L and slope 0.009 mmol/L/min on validation; this newer selection was not at the extended upper boundary. In the test set, it detected 411/449 DKA episodes (91.5%) with 0.761 false alerts/day and a 70 min median lead time. The comparison demonstrates that the original transparent rule was under-optimized relative to the expanded candidate space. It is a post hoc analysis and does not replace the original validation-frozen primary operating point.

#### 3.3.3. Calibration Sensitivity

Primary sigmoid calibration for multimodal XGBoost yielded AUROC 0.989, AUPRC 0.657, Brier score 0.0117, and a validation-selected threshold of 0.45. Isotonic calibration produced similar discrimination (AUROC 0.989; AUPRC 0.648) and selected threshold 0.40. Test episode sensitivity was 89.5% with sigmoid calibration and 90.9% with isotonic calibration; false-alert burdens were 0.456 and 0.534/day, respectively.

Risk-spaced calibration was also examined across the full predicted-probability range and separately for BHB ≥3 observations, where the original quantile-binned figure had insufficient resolution near probability thresholds of 0.40–0.45. The added calibration display is descriptive and should be interpreted with the much smaller conditional sample in mind.

**Figure 6:**
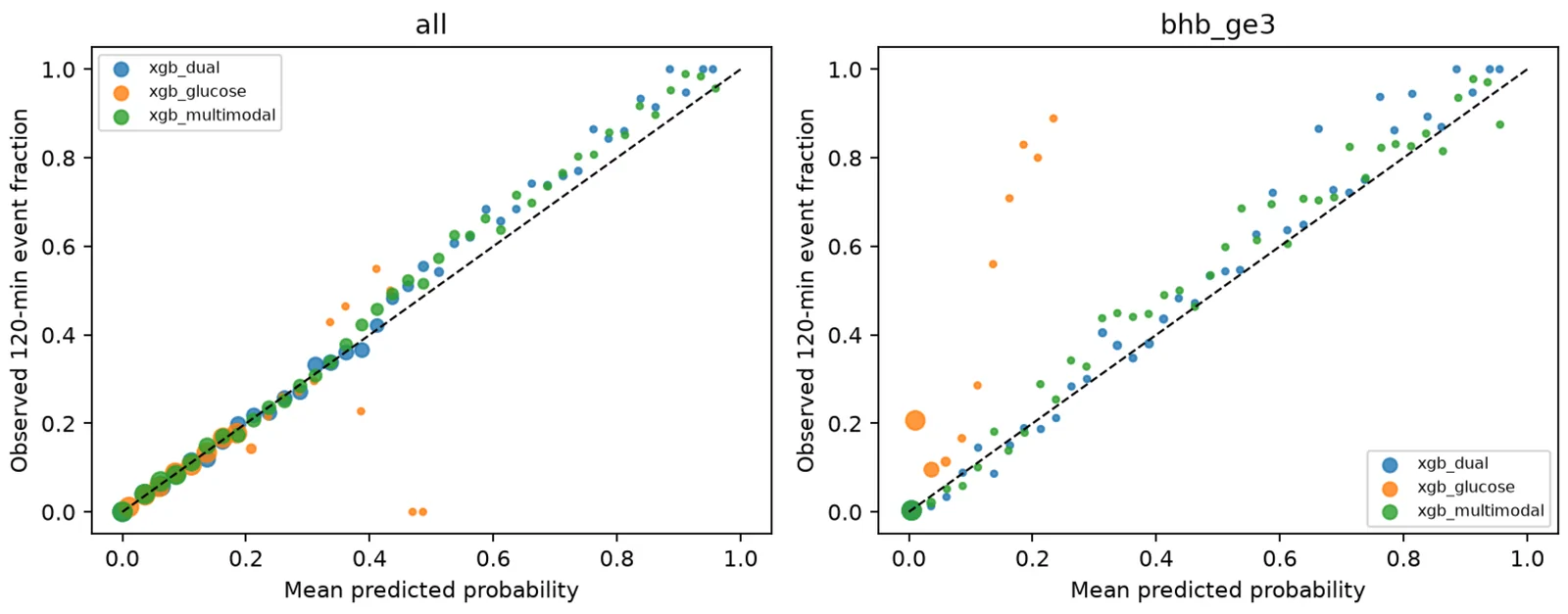
Post hoc risk-spaced calibration analysis on the frozen test set, including high-BHB conditional assessments. Marker area is proportional to the number of observations in each probability bin; larger circles represent more observations. The dashed black diagonal indicates ideal calibration. Unlike the original quantile-binned Figure 4, the displayed probability bins span the range of operational cutoffs.

The post hoc risk-spaced calibration analysis is shown in Figure 6.

The two probability-calibration strategies are compared in Table 8.

#### 3.3.4. Alert-Denominator and Very-Early-Alert Sensitivity Analyses

The independent test set contributed 1680.84 monitored patient-days to the false-alert denominator: 1496.94 days from non-DKA episodes and 183.90 pre-onset days from DKA episodes (10.94% of the denominator). Under the primary 120 min definition, alerts occurring more than 120 min before eventual DKA were counted as false alerts. These accounted for 14.4% of BHB-threshold false alerts, 15.9% of BHB+trend false alerts, 14.7% of BHB-trend-logistic false alerts, 26.3% of dual-XGBoost false alerts, and 40.0% of multimodal-XGBoost false alerts. For multimodal XGBoost, at least one such precursor alert was observed in 157/449 reference-DKA episodes (35.0%). When such alerts were classified separately as early precursors, with 120 min episode sensitivity intentionally unchanged, false-alert burden decreased from 0.456 to 0.274/day for multimodal XGBoost, 0.624 to 0.460/day for dual XGBoost, and 1.060 to 0.891/day for the BHB+trend rule. These values are sensitivity analyses of the outcome definition and do not establish that alerts occurring several hours before DKA would be clinically actionable.

False-alert burden also varied strongly by assigned phenotype. Stable episodes produced zero false alerts for all five strategies. For multimodal XGBoost, burden was 0.504/day in transient ketosis and 0.729/day in hyperglycemia, compared with 3.564/day and 1.756/day, respectively, for the simple BHB threshold. Therefore, the aggregate false-alert rate is dependent on the deliberately enriched synthetic case mix and should not be interpreted as a clinical alarm frequency.

### 3.4. Euglycemic DKA Design Stress Test

The euglycemic-at-onset design-stress subgroup contained 100 independent-test reference-DKA episodes: 55 had been assigned to the classical-DKA simulator phenotype and 45 to the prespecified euglycemic-DKA phenotype. Glucose-only XGBoost detected 31%, whereas dual glucose+BHB and multimodal XGBoost each detected 85% at their independently frozen operating points. The BHB-trend logistic model detected 99%, and both transparent BHB rules detected all 100 episodes. Because low glucose at onset is part of the subgroup construction, weaker glucose-only performance is expected by design. This analysis therefore supports only the design principle that ketone-aware strategies are less dependent on marked hyperglycemia; it is not evidence of clinical effectiveness in euglycemic DKA. The previously shown single-episode example was removed because an illustrative trajectory cannot add independent evidence beyond this prespecified subgroup result.

The euglycemic-DKA design stress test is summarized in Table 9.

### 3.5. Robustness to Missing Measurements and Noise

With 20% random missingness, multimodal episode sensitivity was 89.8% and AUPRC 0.651, similar to the complete-data values of 89.5% and 0.657. Added sensor noise increased episode sensitivity to 92.4% but also increased false-alert burden to 0.654/day. A 30 min contiguous gap reduced sensitivity to 88.0% and AUPRC to 0.635. The gap result therefore represents a measurable performance degradation rather than stable performance under missingness.

The missingness and sensor-noise stress-test results appear in Table 10.

### 3.6. Trajectory-Family Generalization

Family-stratified results and leave-one-family-out retraining were used to test whether XGBoost had simply learned the simulator’s named trajectory templates. When each DKA BHB family was excluded from both training and validation, multimodal XGBoost retained held-out episode sensitivity ranging from 87.1% (pause/resume) to 93.2% (accelerating), with held-out family AUPRC values approximately 0.770–0.885. Dual XGBoost showed a similar absence of catastrophic failure. Generalization to unseen non-DKA families was less uniform: multimodal false-alert burden was 2.18/day when sigmoid-plateau episodes were withheld, 1.48/day for withheld ramp-plateau, and 1.06/day for withheld two-stage-plateau episodes. These findings argue against simple memorization of the five DKA templates, while showing that unseen hard-negative shapes can materially increase alert burden. Full family-level outputs are supplied in the reviewer-sensitivity reproducibility archive.

### 3.7. Distribution-Shift Validation and Explainability

In the separately generated 5000-episode shifted cohort, the frozen multimodal model achieved AUROC 0.981 and AUPRC 0.470, detecting 651/748 reference-DKA episodes (87.0%) with 0.733 false alerts/day and an 85 min median lead time. Dual XGBoost detected 665/748 (88.9%) with 0.980 false alerts/day. The frozen BHB threshold detected 734/748 (98.1%) but generated 2.00 false alerts/day, and the BHB+positive-trend rule detected 730/748 (97.6%) with 1.76 false alerts/day. The shifted cohort therefore preserved the qualitative sensitivity–alert-burden trade-off while showing materially lower AUPRC and reduced ML sensitivity. SHAP analysis of the main test cohort identified the 60 min BHB mean and current BHB as the largest contributors, with smaller contributions from glucose summaries, respiratory rate, and other temporal features.

### 3.8. External Real-World Physiological Benchmarking

The original clinical benchmark was reconstructed exactly from the public SAS data using explicit age, arm, preceding-CGM, and CGM-history restrictions. Adult records comprised 854 BHB–CGM pairs from 165 participants, of which 157 had BHB ≥0.6 and 18 had BHB ≥3.0 mmol/L. The five BHB strata comprised 697, 78, 32, 29, and 18 observations from 161, 53, 26, 19, and 12 participants, respectively. Exactly 100/854 (11.7%) CGM values reached the 401 mg/dL reporting ceiling and were classified as right-censored.

**Figure 7:**
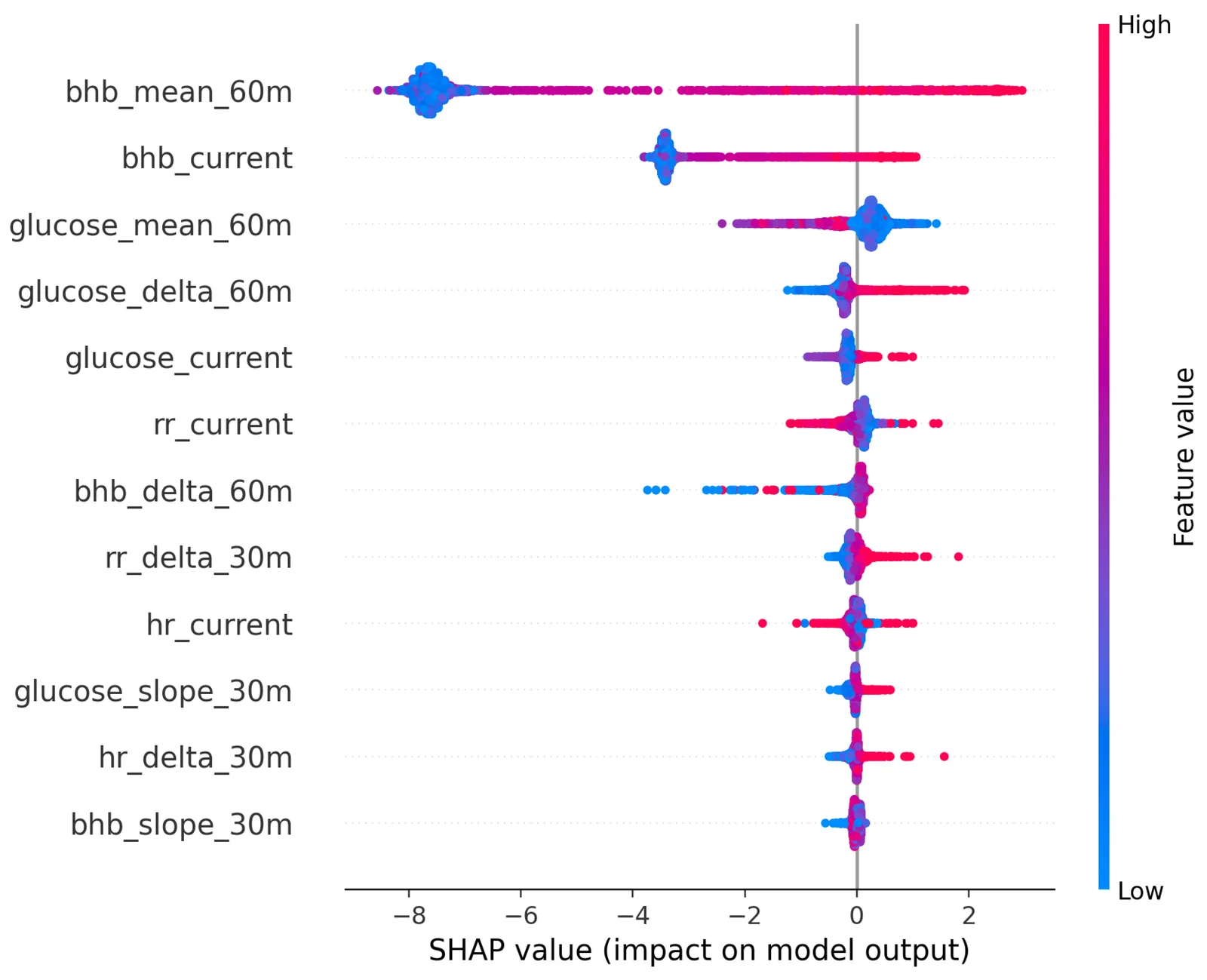
SHAP summary for the multimodal XGBoost model in the independent main-cohort test set.

The global SHAP interpretation is presented in Figure 7.

Observed median glucose exceeded the *all-phenotypes frozen* simulated median in every BHB stratum. The observed-minus-synthetic median differences (95% participant-clustered bootstrap CIs) were 155.7 (115.7–171.7), 243.4 (187.4–251.9), 132.8 (86.8–195.8), 120.8 (98.8–197.8), and 108.7 (16.2–196.2) mg/dL. A capped-data Wasserstein-type distance proxy also indicated marked distributional differences (Table 11). The DKA-assigned synthetic-only comparison likewise exhibited higher real glucose in every stratum, with differences of 156.7, 256.9, 175.5, 165.5, and 156.9 mg/dL. Synthetic 2.5th–97.5th envelopes cannot substantiate agreement when they span nearly the entire CGM reporting range. As a result, the IOBP2 analysis *contradicts*, rather than validates, the simulated central glucose tendencies.

The five-stratum IOBP2 external comparison appears in Table 11.

Across the 854 paired records, 186 consecutive adult BHB intervals lasting 0.5–6 h were available from 76 participants. Only 41 (22.0%) signed slopes were positive; 12 of these 41 fell within the simulator’s originally chosen 0.18–0.35 mmol/L/h interval. Signed slopes, including decreases and unchanged values, are summarized by observation interval in Supplementary Data S1. A hypothetical absolute single-reading BHB uncertainty of δ=0.1 mmol/L would alone allow an apparent absolute slope up to 0.4 mmol/L/h over 0.5 h or 0.2 mmol/L/h over 1 h; this is an illustrative sensitivity assumption, *not* a measured assay precision. IOBP2 had no confirmed DKA events in the primary randomized comparison and therefore cannot calibrate true pre-DKA BHB kinetics.

Signed serial BHB changes are summarized in Table 12.

## 4. Discussion

### 4.1. Principal Findings

The revised analysis supports five main conclusions. First, BHB is entangled with the reference DKA definition: BHB threshold crossing determined reference onset in 61.9% of reference-DKA episodes. Apparent conditional XGBoost discrimination after current BHB had reached 3 mmol/L cannot be interpreted as independently validated clinical information. In this generator, glucose is systematically *lower* in DKA-assigned than non-DKA hyperglycemia hard-negative states at equal high BHB, because BHB begins rising well before latent-severity-driven glucose. The large conditional discrimination therefore reflects simulator-specific phenotype cues. Explicit model counterfactuals and clinical glucose benchmarking support this interpretation.

Second, the apparent advantage of ML depends strongly on operating-point choice. At the originally frozen thresholds, transparent BHB rules had approximately 99% sensitivity with about 1.1–1.2 false alerts/day, whereas multimodal XGBoost had 89.5% sensitivity with 0.456/day. When the strategies were moved to matched regions of the sensitivity–burden frontier, that simple comparison no longer held: multimodal XGBoost required approximately 1.30 false alerts/day to reach about 99% sensitivity, while transparent rules lost most of their sensitivity when constrained to the submitted multimodal burden. Thus, neither transparent rules nor ML uniformly dominated the other.

Third, the apparent multimodal increment depends on glucose and vital-sign processes tied to simulator-assigned phenotypes. Simply drawing HR/RR timing independently within each phenotype left residual class cues and was an inadequate independence control. Under genuinely phenotype-identical vital-sign generation, multimodal and dual AUPRC were nearly indistinguishable (0.6036 vs. 0.6044). Eliminating both glucose and vital-sign phenotype cues also produced near-identical AUPRC (0.5164 vs. 0.5142). These results establish a sensitivity to generator construction and provide no clinical evidence for physiological information contributed by HR/RR or glucose.

Fourth, leave-one-family-out testing did not show catastrophic failure on unseen DKA trajectory families: multimodal sensitivity remained approximately 87–93%. However, some unseen non-DKA plateau-like families generated substantially higher false-alert burdens. This asymmetry is important because clinical non-DKA ketonemia is likely to be more heterogeneous than any finite simulator family set. Distribution-shift performance likewise declined, with multimodal AUPRC falling from 0.657 to 0.470. These results reinforce that the synthetic cohort is a stress-testing environment rather than a validated representation of clinical transportability.

Fifth, alert burden is sensitive to both case mix and outcome definition. Stable episodes generated no false alerts, whereas transient ketosis and hyperglycemia accounted for most non-DKA burden. In addition, 40% of the multimodal model’s primary-definition false alerts occurred more than 120 min before eventual DKA. Reclassifying these as early precursors reduced multimodal burden from 0.456 to 0.274/day without changing 120 min episode sensitivity. This sensitivity analysis illustrates why a fixed prediction horizon can label potentially informative early signals as false, but it does not establish that such signals would be clinically useful or acceptable.

### 4.2. Comparison with Previous Research and Real-World Benchmarking

The present work should be interpreted alongside, rather than as a replacement for, real-data ketone-prediction studies. Cichosz and Bender reported AUROC 0.836 for identifying elevated ketones during hyperglycemia using real continuous-glucose-monitoring data in 138 people with type 1 diabetes [9]. Their later study of 259 participants and more than 49,000 monitoring days achieved AUROC 0.82 and PR-AUC 0.53 using glucose- and insulin-derived features [10]. Those studies used real clinical data and predicted elevated ketone states; the present study used synthetic temporal trajectories, direct BHB observation, and an acid–base-defined DKA endpoint. Accordingly, numerical performance values are not directly comparable. The contribution here is the evaluation architecture: transparent rules and ML models are examined jointly for discrimination, episode sensitivity, lead time, alert burden, missingness, distribution shift, reference circularity, and operating-point dependence.

The reproduced 854-pair IOBP2 benchmark was not physiologically concordant with the frozen simulation. At each of five BHB strata, the observed median glucose was 108.7–243.4 mg/dL above the synthetic median, including in the restricted DKA-assigned comparator. One hundred observations were right-censored by the 401 mg/dL reporting ceiling, and the three highest BHB strata contained only 79 observations. Positive BHB slopes are a selected subset of natural non-DKA ketone variability, not evidence of DKA progression, and apparent short-interval slopes may fall within a plausible but unverified meter-noise band. The absence of an acid–base-confirmed DKA endpoint precludes external clinical assessment of the alarm models. We therefore reject any assertion that IOBP2 validates the simulator’s glucose trajectory or DKA onset kinetics.

### 4.3. Methodological Considerations and Limitations

Reference circularity remains the central limitation. DKA is defined partly by BHB, and the models observe BHB directly. The post hoc onset decomposition and high-BHB conditional analysis quantify the issue but cannot remove it. pH and bicarbonate were hidden from the models, yet their relationship to other simulated channels was still imposed by the data-generating process. Future simulation work should therefore consider alternative endpoints in which the predictor and reference definition are more independent, as well as real datasets with paired BHB and acid–base measurements.

The primary simulator used a shared latent deterioration process for glucose, HR, RR, bicarbonate, and pH, whereas the independently generated BHB trajectories began earlier. This imposed ordering is not physiology-derived and can invert glucose at a given high-BHB state. The non-DKA acid–base floor values of bicarbonate 18.2 mmol/L and pH 7.305 also prevent non-DKA acid–base crossing by construction. Consequently, high-BHB ’hard negatives’ are not independently observed pathophysiological counterexamples. The alternate 24 h aligned-onset exploratory generator is retained only as a sensitivity-development artifact and was not substituted for the frozen 15 h results.

The BHB-rise range and underlying chronology are limited. Klonoff et al. reported an extracted mean rise of 0.0038 mmol/L/min and estimated 14.2 h to BHB 3.0 mmol/L after insulin pump interruption [19]; the synthetic reference-onset median of 10.75 h is earlier, despite the fact that acid–base reference onset is a stricter outcome than BHB alone. Indication-driven intermittent IOBP2 measurements cannot test the simulated rise rates. An assumed δ=0.1 mmol/L per-reading measurement-error sensitivity bound implies an apparent difference-derived slope of as much as 0.4 mmol/L/h across 0.5 h. This is not an assay-validated uncertainty estimate.

The synthetic phenotype mixture was intentionally enriched for hyperglycemia, transient ketosis, and DKA to stress the algorithms and does not represent clinical prevalence. Consequently, aggregate false alerts/day cannot be extrapolated to a hospital population. The phenotype-specific results are provided so that readers can see where burden arose and, if desired, reweight these rates under alternative case-mix assumptions. The primary 120 min alert definition also counts alerts occurring earlier than the horizon as false; the precursor sensitivity analysis demonstrates the effect of that design choice.

Additional limitations include finite trajectory-family libraries, simplified treatment of physiology, absence of treatment actions and feedback, no real-time sensor-specific error model, and no prospective human evaluation. Of 3000 DKA-assigned episodes, 5 did not meet the reference criteria within the main 15 h window, and 2 of 750 did not meet criteria within the shifted 18 h window; no reference label was forced. No nurses or patients interacted with the system, and no usability, workflow, safety, or alarm-acceptability assessment was performed.

### 4.4. Future Nurse-Facing Relevance and Alert Burden

The revised framing deliberately distinguishes future nurse-facing evaluation from a nurse-centered system. No nursing documentation or nurse-generated variables were used, and the current predictors could be consumed by other clinicians or automated systems. The intended future context is monitored adult inpatient care in which glucose, ketone, and vital-sign data are available. No multilevel Green/Yellow/Orange/Red interface or numeric risk-category thresholds are proposed or evaluated in this study.

Alert burden remains clinically important, but false alerts per simulated patient-day should not be equated with alert fatigue. Reviews of ICU alarms and clinical decision support emphasize that fatigue depends not only on alert quantity but also relevance, workflow, response, cognitive load, and desensitization [14]. Whether approximately one alert/day, 0.45/day, or any other burden is acceptable cannot be determined from simulation. A future study should use standardized scenarios and prospective observation to measure recognition time, escalation decisions, workload, trust, response rates, and perceived alert relevance with nurses and other clinicians.

### 4.5. Future Research

The immediate priority is external validation using real paired glucose–BHB time series with independently observed acid–base-defined DKA onset, including both sustained ketonemia that progresses to acidosis and hard-negative ketonemia that resolves. Continuous or high-frequency BHB data would allow the frozen rules and temporal models to be evaluated without redefining the input representation. Subsequent work should include human-factors co-design and evaluation with nurses, diabetes specialists, physicians, and informatics teams before any clinical deployment. The simulation generator, frozen configuration, primary outputs, peer-review analysis scripts, and derived reviewer-sensitivity outputs are supplied with the revised Supplementary Materials to facilitate independent reproduction and challenge.

## 5. Conclusions

This simulation-only evaluation of a DKA early-warning framework identifies operating-point trade-offs between transparent BHB rules and XGBoost models and provides reproducible tests of algorithmic fragility. The original BHB+trend rule achieved 98.9% sensitivity at 1.060 false alerts/day and the multimodal model 89.5% at 0.456/day on synthetic data, but these represent different sensitivity–burden operating points. An extended comparator grid changed the rule result, and model counterfactuals showed that the apparent incremental XGBoost discrimination arose in part from phenotype-specific glucose and vital-sign generation. In the current simulator, glucose was lower in DKA than hard-negative hyperglycemia at comparable BHB; conditional high-BHB model discrimination cannot establish physiological value beyond the BHB measurement.

Reconstruction of 854 paired BHB–CGM observations from 165 IOBP2 adults showed that measured glucose medians exceeded simulated medians by 108.7–243.4 mg/dL in every BHB stratum, exposing a major representation mismatch. The available human ketone slopes did not include DKA events and cannot test DKA kinetics. The deliverable is therefore a computational stress-testing framework with documented structural limitations, not a clinically validated DKA predictor, validated nurse-facing interface, or demonstration of ML superiority. This framework remains a simulation-based methodological development intended for future nurse-facing evaluation. It has not undergone external clinical validation, and its predictive performance, warning lead times, and alert burden cannot be generalized to real patients or clinical care. Although the IOBP2 dataset provided an independent physiological benchmark, it did not validate DKA prediction performance. Prospective studies using patient-level glucose, ketone, and acid–base measurements, followed by nurse-facing usability and clinical safety evaluation, are required before clinical implementation.

## Figures and Tables

**Table 1:** Synthetic cohort composition by assigned phenotype.

Simulated Phenotype	Number	Percentage
Stable diabetes	9000	45.0%
Non-ketotic hyperglycemia	5000	25.0%
Transient/non-DKA ketosis	3000	15.0%
Classical DKA progression	2700	13.5%
Euglycemic DKA progression	300	1.5%
Total	20,000	100.0%

**Table 2:** Simulated physiological variables and prespecified behavior.

Variable	Baseline/Non-DKA Behavior	DKA Deterioration Behavior
Glucose	90–180 mg/dL baseline; hyperglycemia may rise substantially	Classical DKA: broad hyperglycemic rise; euglycemic episodes constrained <200 mg/dL at onset
BHB	0.10–0.55 mmol/L baseline; hyperglycemia up to 3.4; transient ketosis 0.8–4.8	Start 0.25–0.75 mmol/L; heterogeneous families with rates mainly 0.18–0.35 mmol/L/h
Heart rate	62–92 beats/min; non-DKA stress changes permitted	Prespecified simulation increase of 0–42 beats/min
Respiratory rate	12–18 breaths/min; non-DKA changes permitted	Prespecified simulation increase of 0–16 breaths/min
Bicarbonate/pH	Non-DKA constrained outside DKA reference thresholds	May cross ${\mathrm{HCO}}_{3}^{-}<18$ mmol/L and/or pH <7.30; hidden from predictors
Stochastic variation	AR(1) correlated noise	AR(1) correlated noise plus independently sampled timing offsets

**Table 3:** Predictor configurations evaluated.

Predictor Set	Variables/Features
Glucose-only XGBoost	Current glucose; 30 min slope; 60 min mean; 60 min change
Dual glucose–BHB XGBoost	Glucose features plus current BHB; 30 min BHB slope; 60 min BHB mean; 60 min BHB change
Multimodal XGBoost	Dual features plus current HR/RR and 30 min HR/RR changes
BHB-trend logistic	Current BHB and 30 min BHB slope

**Table 4:** Final model and calibration specifications.

Model/Component	Final Specification
BHB-trend logistic	Median imputation; standardization; balanced L2 logistic regression
XGBoost	450 trees; depth 5; learning rate 0.04; subsample 0.85; colsample 0.85; min_child_weight 5; λ=2.0
Class weighting	XGBoost scale_pos_weight = 43.902 from the final training partition (2,208,444 negative and 50,304 positive time points); logistic regression used class_weight = balanced
Calibration	Platt/sigmoid primary; isotonic sensitivity analysis; both fitted on validation only

**Table 5:** Independent test-set discrimination and calibration performance. Calibration intercept and slope confidence intervals use episode-cluster-robust standard errors.

Model	AUROC (95% CI)	AUPRC (95% CI)	Brier (95% CI)	Cal. Intercept (95% CI)	Cal. Slope (95% CI)
Glucose-only XGBoost	0.758 (0.740–0.776)	0.105 (0.092–0.124)	0.0207 (0.0190–0.0225)	−0.084 (−0.312–0.144)	0.974 (0.911–1.036)
Glucose + BHB XGBoost	0.987 (0.985–0.988)	0.604 (0.569–0.640)	0.0127 (0.0119–0.0137)	0.122 (−0.020–0.265)	1.075 (0.998–1.152)
Multimodal XGBoost	0.989 (0.988–0.990)	0.657 (0.630–0.695)	0.0117 (0.0107–0.0127)	0.168 (0.029–0.307)	1.074 (1.000–1.149)
BHB-trend logistic	0.969 (0.965–0.972)	0.262 (0.235–0.294)	0.0194 (0.0181–0.0211)	0.052 (−0.205–0.310)	1.010 (0.943–1.077)

**Table 6:** Pre-onset independent-test glucose among observations with current BHB ≥3.0 mmol/L, by assigned phenotype. Repeated rows are not independent clinical people.

Assigned Phenotype	Rows	Episodes	Median Glucose	IQR, mg/dL
Classical DKA	2666	150	152.9	123.8–187.6
Euglycemic DKA	303	17	149.8	123.5–165.3
Non-DKA hyperglycemia	2389	85	304.5	259.7–360.3
Non-DKA transient ketosis	4907	156	196.0	165.1–227.3

**Table 7:** Early-warning performance at validation-frozen operating points.

Strategy	Sensitivity (95% CI)	Detected	Lead Time (95% CI)	False Alerts/Day (95% CI)
Glucose-only XGBoost (minimum tested threshold 0.05)	78.8% (descriptive)	354/449	80 min	4.890
BHB threshold 2.75 mmol/L	98.9% (97.8–99.8)	444/449	75 min (70–80)	1.193 (1.074–1.324)
BHB + positive-trend AND rule	98.9% (97.8–99.8)	444/449	70 min (69–75)	1.060 (0.949–1.160)
BHB-trend logistic	98.7% (97.4–99.6)	443/449	70 min (65–75)	1.192 (1.072–1.338)
Dual glucose+BHB XGBoost	91.8% (88.9–94.3)	412/449	80 min (80–85)	0.624 (0.551–0.702)
Multimodal XGBoost	89.5% (86.8–92.2)	402/449	85 min (80–90)	0.456 (0.402–0.524)

**Table 8:** Sensitivity of alert performance to probability-calibration method.

Calibration	AUPRC	Episode Sensitivity	False Alerts/Patient-Day
Platt/sigmoid (primary)	0.657	89.5%	0.456
Isotonic (sensitivity analysis)	0.648	90.9%	0.534

**Table 9:** Performance in the prespecified euglycemic-at-onset design stress test.

Method	Sensitivity	Detected/100	Median Warning Lead Time
Glucose-only XGBoost	31.0%	31/100	30 min
BHB threshold rule	100.0%	100/100	87.5 min
BHB-trend logistic	99.0%	99/100	80 min
Dual glucose+BHB XGBoost	85.0%	85/100	75 min
Multimodal XGBoost	85.0%	85/100	85 min

**Table 10:** Robustness of multimodal XGBoost to missingness and measurement noise.

Test Condition	AUROC	AUPRC	Sensitivity/False Alerts per Patient-Day
Complete data	0.989	0.657	89.5%/0.456
20% random missingness	0.989	0.651	89.8%/0.438
30 min contiguous gap	0.986	0.635	88.0%/0.416
Added sensor noise	0.987	0.621	92.4%/0.654

**Table 11:** Adult IOBP2 versus frozen all-phenotypes synthetic glucose by BHB stratum. CIs resample clinical participants 2000 times; distributional distances use right-censored CGM values as descriptive proxies.

BHB	Pairs/People	At 401	Real Median	Synth. Median	Difference (95% CI), mg/dL	Dist. Proxy
<0.6	697/161	41	292.0	136.3	155.7 (115.7–171.7)	123.4
0.6–<1.0	78/53	37	392.5	149.1	243.4 (187.4–251.9)	163.9
1.0–<1.5	32/26	8	312.0	179.2	132.8 (86.8–195.8)	93.1
1.5–<3.0	29/19	10	324.0	203.2	120.8 (98.8–197.8)	102.6
≥3.0	18/12	4	310.5	201.8	108.7 (16.2–196.2)	79.2

**Table 12:** Signed serial BHB changes among IOBP2 adults across eligible 0.5–6 h intervals.

Interval (Hours)	Intervals	People	Median Signed Slope	Positive Fraction
0.5–1.0	27	22	−0.109	25.9%
1.0–2.0	96	52	−0.062	18.8%
2.0–3.0	39	26	−0.141	12.8%
3.0–4.0	14	9	0.000	35.7%
4.0–6.0	10	10	0.029	60.0%

## Data Availability

Source IOBP2 clinical records are available via the Jaeb Center for Health Research public dataset [21], subject to source terms of use; no participant-linked clinical rows are redistributed. The executable local reconstruction and aggregate-derivation scripts and revised aggregate Supplementary Data S1 accompany this submission. The synthetic study source, frozen simulation configuration, trained model artifacts, test aggregate results, and counterfactual code are supplied in Supplementary Files S2 and S3. The public Zenodo all-versions DOI is https://doi.org/10.5281/zenodo.23334512.

## Supplementary Materials

The following supporting information can be downloaded at https://www.mdpi.com/article/10.3390/healthcare14213790/s1, Supplementary Data S1 provides the reconstructed aggregate IOBP2 adult benchmark, synthetic-comparator denominators, five-stratum participant-clustered comparisons, signed serial BHB kinetics, and explicitly hypothetical noise-bound sensitivity. Supplementary File S2 contains the primary frozen simulator, configuration, original trained outputs, and code documentation. Supplementary File S3 provides second-round response analyses, new counterfactual results, calibration plots, expanded comparator grid, and executable IOBP2 linkage/reanalysis scripts. The scripts require the public Jaeb IOBP2 dataset to reproduce participant-linked steps locally; no participant-level records are redistributed. Figures S1 and S2 are retained as contextual legacy descriptive displays; the revised primary benchmark table replaces envelope-coverage claims with median differences and uncertainty.

## Abbreviations

DKA	Diabetic ketoacidosis
BHB	Beta-hydroxybutyrate
ML	Machine learning
AUROC	Area under the receiver-operating-characteristic curve
AUPRC	Area under the precision–recall curve
CGM	Continuous glucose monitoring
HR	Heart rate
RR	Respiratory rate
SHAP	Shapley additive explanations
